# Binding of the kringle‐2 domain of human plasminogen to streptococcal PAM‐type M‐protein causes dissociation of PAM dimers

**DOI:** 10.1002/mbo3.1252

**Published:** 2021-11-30

**Authors:** Olawole Ayinuola, Yetunde A. Ayinuola, Cunjia Qiu, Shaun W. Lee, Victoria A. Ploplis, Francis J. Castellino

**Affiliations:** ^1^ W. M. Keck Center for Transgene Research University of Notre Dame Notre Dame Indiana USA; ^2^ Department of Chemistry and Biochemistry University of Notre Dame Notre Dame Indiana USA; ^3^ Department of Biological Sciences University of Notre Dame Notre Dame Indiana USA

**Keywords:** analytical ultracentrifugation, circular dichroism, human plasminogen, isothermal calorimetry, kringle domains, streptococcal plasminogen binding M‐protein

## Abstract

The direct binding of human plasminogen (hPg), via its kringle‐2 domain (K2_hPg_), to streptococcal M‐protein (PAM), largely contributes to the pathogenesis of Pattern D Group A *Streptococcus pyogenes* (GAS). However, the mechanism of complex formation is unknown. In a system consisting of a Class II PAM from Pattern D GAS isolate NS88.2 (PAM_NS88.2_), with one K2_hPg_ binding a‐repeat in its A‐domain, we employed biophysical techniques to analyze the mechanism of the K2_hPg_/PAM_NS88.2_ interaction. We show that apo‐PAM_NS88.2_ is a coiled‐coil homodimer (M.Wt. ~80 kDa) at 4°C–25°C, and is monomeric (M.Wt. ~40 kDa) at 37°C, demonstrating a temperature‐dependent dissociation of PAM_NS88.2_ over a narrow temperature range. PAM_NS88.2_ displayed a single tight binding site for K2_hPg_ at 4°C, which progressively increased at 25°C through 37°C. We isolated the K2_hPg_/PAM_NS88.2_ complexes at 4°C, 25°C, and 37°C and found molecular weights of ~50 kDa at each temperature, corresponding to a 1:1 (m:m) K2_hPg_/PAM_NS88.2_ monomer complex. hPg activation experiments by streptokinase demonstrated that the hPg/PAM_NS88.2_ monomer complexes are fully functional. The data show that PAM dimers dissociate into functional monomers at physiological temperatures or when presented with the active hPg module (K2_hPg_) showing that PAM is a functional monomer at 37°C.

## Graphical abstract

PAM‐type M‐proteins on the surface of *Streptococcus pyogenes* are monomeric (not the usually depicted dimers) at functional temperatures and bind human plasminogen as monomers.

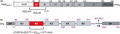

## 1 INTRODUCTION

Group A *Streptococcus* (GAS) is a Gram‐positive β‐hemolytic human‐selective pathogen that causes a myriad of mild‐severe infectious diseases, including pharyngitis, impetigo, necrotizing *fasciitis*, acute rheumatic fever, and streptococcal toxic shock syndrome (Carapetis et al., 2005; Cole et al., 2011; Walker et al., 2014). GAS infections are thus perceived as serious global health concerns in terms of both mortality and morbidity (Carapetis et al., 2005; Lee et al., 2009; Seckeler & Hoke, 2011).

The virulence of GAS is partly dictated by the nature of its M‐protein (*emm* gene), a multi‐copy surface virulence determinant expressed by GAS that covalently binds to the cell wall of Gram‐positive bacteria and extends beyond the capsular cell surface as a hair‐like projection. This protein is the basis of *emm* gene serotyping for the >250 strains of GAS that have been identified (Fischetti, 1989; Phillips et al., 1981). Functionally, M‐protein is a versatile virulence factor that participates in several resistance mechanisms aimed at the elimination of GAS. These extended surface M‐protein appendages initially mediate strong attachment of bacteria to keratinocytes and epithelial cells to initiate infection (Courtney et al., 1992; Cue et al., 1998; Ellen & Gibbons, 1972). Some serotypically distinct M‐proteins, e.g., M1‐, M3 ‐, and M6‐ proteins, expressed by the *emm1*, *emm3*, and *emm6* genes, respectively, directly bind to extracellular matrix components (ECM), such as fibronectin, to promote bacterial colonization (Cue et al., 2001; Schmidt et al., 1993). In addition to the mediation of bacterial adhesion to host cells, blockage of the complement pathways (Agrahari et al., 2013; Buffalo et al., 2016; Prasad et al., 2015) and upregulation of the fibrinolytic system on the GAS surface (Glinton et al., 2017) are other major functions of some M‐proteins.

Plasminogen‐binding Group A streptococcal M‐protein (PAM) is a specialized M‐protein found on skin‐tropic Pattern D GAS strains (Bessen & Lizano, 2010) that directly binds to human plasminogen (hPg) with nM‐scale affinity, and, accordingly, accumulates hPg on the GAS cell surface. There are several sequence variations of PAM‐type M‐proteins on different strains of Pattern D GAS (Qiu et al., 2018), but all bind tightly to hPg and stimulate hPg activation by the GAS‐secreted streptokinase (SK2b) that is coinherited with PAMs on Pattern D strains (Zhang et al., 2012). These steps result in activation of hPg bound to GAS cells in this manner and thus generate a functional protease, plasmin (hPm), on the GAS surface. These proteolytically competent cells degrade the fibrin formed as a host response to infection that encapsulates invading GAS cells, thereby first promoting the release of the bacteria and, ultimately, dissemination of these pathogens by digesting cellular tight junction proteins and degrading the extracellular matrix (Loof et al., 2014).

Structurally, all known PAMs are domain‐assembled proteins composed consecutively of a 41‐residue NH_2_‐terminal signal peptide, a hypervariable region (HVR), A‐, B‐, C‐, and D‐domains, a Pro/Gly‐rich region, a LPXTG motif recognized by sortase A, a COOH‐terminal transmembrane anchor, and a very short C‐terminal extracellular region (Fischetti et al., 1988; Smeesters et al., 2010). In Pattern D strains, PAM subdomains, viz., the a1 and/or a2 repeats of its A‐domain, specifically bind to the kringle‐2 module of hPg (K2_hPg_; Berge & Sjobring, 1993; Rios‐Steiner et al., 2001). According to the amino acid sequence and the number of tandem repeats in the PAM A‐domain, we previously categorized PAMs into three classes: Class I and Class III (both containing a1‐ and a2‐repeats) and Class II (a2‐repeat only; Qiu et al., 2018). Each class of PAM tightly associates with hPg in the low‐nM range. We hence established a PAM structure‐function model of solution phase PAM to elucidate the significant participation of A‐domain α‐helices in hPg‐binding (Qiu et al., 2020).

The C‐domain of PAM contains the most abundant α‐helices among all domains. These regions of PAM drive parallel coiled‐coil dimerization *via* hydrophobic clustering that is mediated by Leu‐ and Val‐containing heptad repeats (Fischetti et al., 1988, 1990; Qiu et al., 2018). This non‐covalent dimerization mode is stable at ≤25°C but is disrupted by a small increase in temperature in the physiological range (Qiu et al., 2019). At 37°C, most PAM dimers dissociate, leading to a large portion of monomers, irrespective of the PAM classifications (Qiu et al., 2019). This suggests that the dimer is not highly stable. Nonetheless, it is thus far unknown whether K2_hPg_‐binding is another event leading to the dissociation of PAM dimers. This investigation addresses this uncertainty.

## 2 MATERIALS AND METHODS

### 2.1 Protein expression and purification

M‐protein (PAM_NS88.2_) was cloned from GAS isolate NS88.2 (*emm* serotype 98.1), obtained from Dr. Mark Walker, Queensland, AU. The coding sequence of mature full‐length recombinant (r) PAM_NS88.2_ (Pattern D, vir type 17.4, *emm* 98.1) (McKay et al., 2004) begins at the first amino acid residue in the HVR (D^1^ of the protein after cleavage of a 41‐residue signal peptide, and terminates at the last residue of the Pro/Gly‐rich region (Q^344^), slightly upstream of the L^345^PSTG sortase A (srtA) cleavage motif region were excluded from construction of *pam* expression plasmids (Chandrahas et al., 2015; Glinton et al., 2017; Qiu et al., 2018, 2019; Zhang et al., 2012).

AGL55, cloned from the PAM_NS88.2_ gene, encompasses A^54^‐Y^108^ of PAM_NS88.2_. This construct contains 14 COOH‐terminal residues of the HVR, the entire hPg binding a‐repeat (D^68^‐E^85^), and 24 NH_2_‐terminal residues of the downstream B‐domain (Qiu et al., 2018, 2019).

Recombinant AGL55 and PAM_NS88.2_ contained NH_2_‐ and COOH‐terminal His_6_ tags, respectively, for ease in purification by Ni^+^‐based affinity chromatography. Detailed procedures with respect to expression in *Escherichia coli* BL21 (DE3) cells and purification of PAM proteins and peptides have been reported (Qiu et al., 2018; Yuan et al., 2017).

K2_hPg_ (E^164^E‐C^166^‐C^243^ of mature hPg) was cloned from the *hpg* gene, expressed in *Pichia pastoris*, and purified on Lys‐Sepharose (Nilsen et al., 1999). There were several additional residues at the N‐ and C‐termini of K2_hPg_ which were derived from the nature of the processing of the expression plasmid in *P*. *pastoris* cells.

### 2.2 Isolation of the K2_hPg_/PAM_NS88.2_ complexes

K2_hPg_ and PAM_NS88.2_, dissolved in 50 mM sodium phosphate/100 mM NaCl, pH 7.4, were mixed at a molar ratio of 1.5 (27 µM):1 (18 µM) (m:m, based on monomer M. Wt.) and incubated for 10 min with gentle swirling. The mixture was then applied to a Sephadex G‐50 column (1 × 70 cm) pre‐equilibrated with the sample buffer at either 4°C, 25°C, or 37°C. Elution of the complex was accomplished using the sample buffer at a flow rate of 1 ml/min.

### 2.3 Isothermal titration calorimetry (ITC)

All titrations were conducted in a VP‐ITC Microcalorimeter (Malvern Panalytical, UK) at the designated temperatures. The proteins were dissolved in 50 mM sodium phosphate/100 mM NaCl, pH 7.4. The reference cell was filled with H_2_O and kept closed during the entire experiment. The sample cell was loaded with PAM_NS88.2_ (6 µM based on monomer M. Wt. or 3 µM based on the dimer M. Wt.) or AGL55 (30 µM) solution, while the syringe contained K2_hPg_ (120 or 600 µM). The volume of each injection was 3 μL, and the flow rate was 0.5 μl/s. K2_hPg_ was injected into the PAM solution ~30 times, depending on the endpoint of the titration. A 180‐s spacing period between injections was set for the equilibration of heat changes. During the entire titration, the syringe stirred at a rate of 329 rpm. The differential power (DP) was initially set at 10 μcal/sec. When the actual DP stabilized in the range of 10 ± 1 μcal/s, the titration automatically started and the data were recorded in real‐time. Raw thermograms were integrated into NITPIC (Keller et al., 2012; Scheuermann & Brautigam, 2015), generating isotherms which were exported to, and analyzed by the Sedphat hetero‐association model (A + B → AB) (Zhao et al., 2015).

### 2.4 Analytical ultracentrifugation (AUC)

Sedimentation velocity (SV) and sedimentation equilibrium (SE) experiments were conducted using a Beckman Optima XL‐I analytical ultracentrifuge in the absorbance optics mode. SV experiments were conducted for 17–44 h depending on the temperature (4, 15, 25°C, or 37°C) with a rotor speed of 170,700 *g* using 1.2 cm two‐channel centerpieces. Prior to the loading step, all proteins were dissolved in 50 mM sodium phosphate/100 mM NaCl, pH 7.4 (sample buffer). PAM_NS88.2_ was diluted to A_230 nm_ of ~0.15, 0.3, and 0.6. In each assembled cell, 420 μl sample buffer, was injected as the reference into the left channel, and 400 μl of the protein solution was loaded into the right channel. Scans were recorded every three mins and 300–700 scans were collected for each SV experiment. The data were analyzed by Sedfit (version 14.1) using a continuous *c(s)* distribution model (Schuck et al., 2014). The values of viscosity and density of the buffer solutions were obtained using Sednterp (http://www.rasmb.org/sednterp). The normalized c(s) *vs*. S_20_,w plots were generated using public domain software, GUSSI version 1.4.2 (https://www.utsouthwestern.edu/labs/mbr.software).

For AUC‐SE experiments, PAM_NS88.2_ (A_280nm_ ~0.125) and purified PAM_NS88.2_/K2_hPg_ (A_280 nm_ ~0.3) were loaded into the sample well of a two‐sector centerpiece. The sample channel contained 150 μL protein while the reference channel was loaded with 160 μl sample buffer. All experiments were conducted at 4°C, 25°C and 37°C. Scans were recorded hourly at 26,100 *g* and 32,200 *g* until equilibrium was achieved, as evaluated by the lack of change of the concentration gradient with time. The equilibrium data were analyzed for molecular weight using Optima XL‐A/XL‐I software (Beckman Coulter). Buffer densities and viscosities were determined using Sednterp (http://www.rasmb.org/sednterp).

### 2.5 Circular dichroism (CD)

Far‐UV CD spectral measurements in the wavelength range of 195–250 nm were conducted using a Jasco J‐815 circular dichroism spectropolarimeter and a 1 mm path‐length cuvette. The buffer used was 50 mM sodium phosphate, pH 7.4, at 4°C, 25°C, and 37°C. To observe the influence of K2_hPg_ on the 2°structure of PAM_NS88.2_ and AGL55, the CD spectra of these proteins/peptides were mixed with excess K2_hPg_ and the spectra were collected. The spectrum of K2_hPg_, alone, which is non‐helical, was similarly obtained and subtracted from that of the complex. An average of three replicate scans, from which an average buffer scan was subtracted, were collected. For the AGL55 experiments, the concentration of AGL55 was 10 µM, and that of K2_hPg_, when present, was 12.5 µM. For the PAM_NS88.2_ experiments, the concentration of PAM_NS88.2_ was 0.3 µM and that of K2_hPg_, when present, was 1.0 µM.

Mean residue ellipticity ([*θ*]) of the proteins/peptides were calculated using: [*θ*] = (*θ*_obs_ × MRW)/(*l*  ×  c), where *θ*_obs_ is the observed signal in mdeg, MRW is the mean residue weight in g/mol, *l* is the path length in mm, and c is the protein concentration in mg/ml (Greenfield, 2006). The fractional α‐helical content (*f*_H_) was estimated from: [*θ*]_222 nm_ = −30,300*f*_H_ −2,340, in which *f*_H_ refers to the fraction of helices in the protein (Chen et al., 1972). Very little interference was obtained from K2_hPg_ at this wavelength since K2_hPg_ does not contain α‐helical regions (Wang et al., 2010).

### 2.6 Size exclusion chromatography/multi detection system

An Agilent 1260 II HPLC system (Agilent Technologies), which consists of a variable wavelength detector, a quaternary pump, an online vacuum degasser, and an autosampler, was used in tandem with a multiangle light scattering (MALS) detector (DAWN‐HELEOS; Wyatt Technology), and a differential refractive index detector (Optilab^®^ T‐Rex; Wyatt Technology). The mobile phase was 50 mM Na‐phosphate/100 mM NaCl, pH 7.4 (PBS). Prior to injection, the protein samples were passed through a membrane filter (pore size 0.1 μm; Supelco Analytical). The sample volume injected into the channel was 100 μl, and the flow rate was set at 0.5 ml/min. Data acquisition and evaluation were accomplished using Astra 7 software (Wyatt Technology Corporation).

The stoichiometry of binding between the AGL55 and K2_hPg_ in the two‐component complex was determined using the Protein Conjugate Analysis Method in Astra 7 software (Wyatt Technology). This method, based on the unique specific refractive index increments (d*η*/d*c*) and extinction coefficients of each protein enables accurate determination of the components of the complex along molecular mass distributions.

### 2.7 hPg activation assays

The assays were performed at 25°C, and 37°C in 96‐well microtiter plates using the chromogenic substrate, H‐D‐Val‐L‐Leu‐L‐Lys‐p‐nitroanilide (S2251; Chromogenix), to monitor the generation of hPm with time. A typical assay mixture contained hPg (200 nM), various concentrations of PAM_NS88.2_ and S2251 (0.25 mM) in 10 mM Na‐Hepes/150 mM NaCl, pH 7.4. The reaction was accelerated by adding SK2b (5 nM). The release of p‐nitroaniline from S2251, as catalyzed by hPm, was continuously monitored at 405 nm for up to 120 min. The initial velocities were calculated from slopes of plots of *A*_405nm_ vs *t* ^2^.

## 3 RESULTS

### 3.1 Binding of AGL55 to K2_hPg_

**FIGURE 1:**
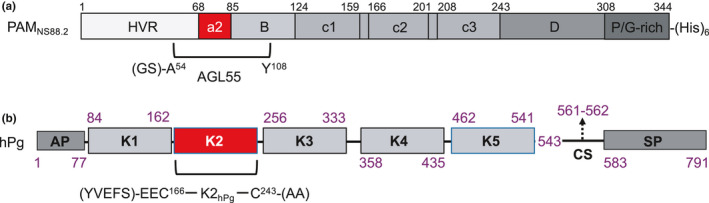
Schematic depiction of the proteins. (A) *PAM_NS88_*_._ *_2_*. The genomic DNA of PAM_NS88.2_ was cloned from GAS isolate NS88.2, inserted into the expression plasmid, pET‐28a, and expressed in *E*. *coli* BL21 cells. The protein was purified by Ni^+^‐based affinity chromatography. AGL55 was constructed as [MGSS(H)_6_GB1‐(thrombin cleavage peptide‐LVPR’GS)‐AGL55]. After expression in *E*. *coli* and purification using Ni^+^‐based affinity chromatography, the peptide was cleaved by thrombin (at R‘G) and repurified by the same column as the column pass‐through. GS‐AGL55 was the remaining peptide. (B) *K2_hPg_*, consisting of residues C^166^‐C^243^, preceded by EE from hPg, was cloned from the *hPg* gene, inserted into expression plasmid pPIC9K, expressed in *P*. *pastoris* cells, and purified by Lys‐Sepharose affinity chromatography. The (YVEFS) residues at the N‐terminus, as well as the (AA) at the C‐terminus, originated from the poly‐linker sites of the expression plasmid

It was previously found that two monomeric A‐domain peptides, containing a1‐ and a2‐repeats, derived from Class I/III PAMs, viz., a 38‐mer from PAM_NS455_ (VKK38_NS455_) (Yuan et al., 2017) and a 50‐mer from PAM_AP53_ (VEK50_AP53_) (Yuan et al., 2019), respectively, both bound K2_hPg_ with a stoichiometry of 2:1 (m:m) at 25°C, indicating that the two a‐repeats (a1 and a2) housed within the sequences of these PAM peptides are simultaneously functional. The equivalent monomeric 55‐mer (AGL55) from the Class II PAM, PAM_NS88.2_ (Figure 1A) contains one a‐repeat and we expect that this peptide will contain one binding site for K2_hPg_ (Figure 1B).

**FIGURE 2:**
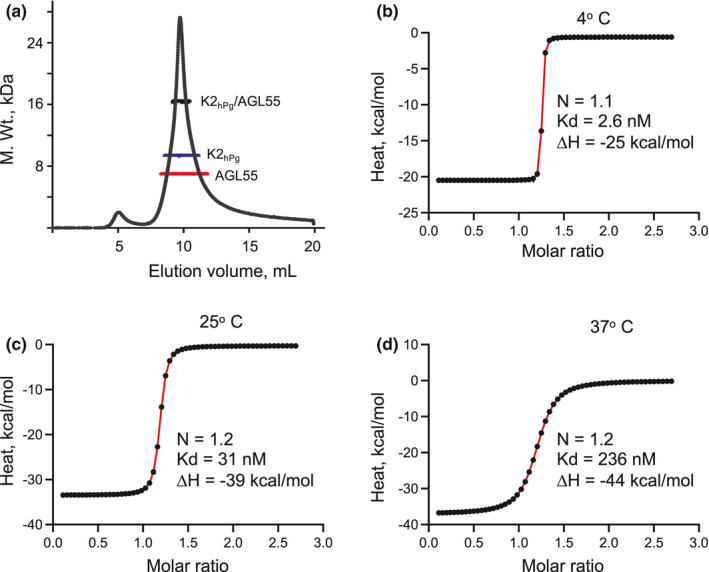
Complex formation of AGL55 with K2_hPg_. (A) RI fractograms representing the K2_hPg_/AGL55 complex, together with the M. Wt. versus elution volume; black for the K2_hPg_/AGL55 complex, blue for the K2_hPg_ constituent of the complex, and red for the AGL55 constituent of the complex. The protein complex (100 µl) complex was injected onto a Wyatt ‐ 030S5 column (7.6 mm × 300 mm, 5 µM, 300 Å), equilibrated, and eluted with PBS, pH 7.4, at a flow rate of 0.5 ml/min. The M. Wt. of the complex of 16.2 kDa is consistent with a 1:1 complex of K2_hPg_ (M. Wt. of 10.2 kDa) and AGL55 (M. Wt. 6.6 kDa). (B–D). K2_hPg_ was titrated into AGL55 at 4°C (b), 25°C (C), and 37°C (D). The heat changes, measured by ITC, accompanying each injection are plotted against the molar ratio of K2_hPg_ to AGL55. The data points are in black and the best‐fit lines for these points are shown in red. The experimental data were best‐fit using the hetero‐association model of K2hPg and AGL55 in SEDPHAT, which provided values of N, Kd, and ΔH for the K2_hPg_/AGL55 interaction

To initially determine whether a K2_hPg_/AGL55 complex was formed at 25°C, a size exclusion chromatography (SEC) system was employed, which consisted of a multi‐detection system (ultraviolet, refractive index, multi‐angle light scattering) (Rebolj et al., 2012). From the deconvoluted fractogram (Figure 2A), all three components, viz., AGL55, K2_hPg_, and the 1:1 (m:m) complex of the two components are present, suggesting that a tight complex is formed. Further, from ITC experiments one strong binding site for K2_hPg_ on PAM_NS88.2_ is found at temperatures of 4°C, 25°C, and 37°C (Figure 2B–D) with Kd and ΔH values that increase with temperature as expected from the exothermic nature of the binding interaction. At all temperatures, the binding stoichiometry is 1:1 (m:m).

**FIGURE 3:**
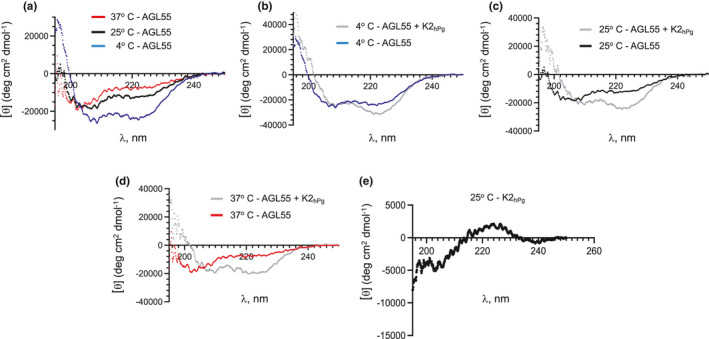
Effect of K2_hPg_ on the secondary structure of AGL55 at various temperatures. Far‐UV mean residue ellipticities *[θ]* of each protein and peptide as a function of wavelength as obtained from the CD signals. (A) Apo‐AGL55 at 4°C, 25°C, and 37°C demonstrate the loss of a a‐helix in AGL55 at 37°C. The graph shown is an overlay of individual spectra at 4°C, 25°C, and 37°C. (B–D) The complexes of K2_hPg_/AGL55 at 4°C (B), 25°C (C), and 37°C (D) as compared to AGL55 alone (from A) at these same temperatures. (E) K2_hPg_, alone, at 25°C showing the lack of α‐helices in this domain. The concentration of AGL55 was 10 mM and the K2_hPg_ was 12.5 mM when present. The buffer was 50 mM Na‐phosphate, pH 7.4 at each temperature

The interaction of PAM and hPg is dependent on an α‐helix within the a‐repeats of PAM for optimal organization of the amino acid side chains required for binding (Qiu et al., 2020; Rios‐Steiner et al., 2001; Wang et al., 2010). Thus, we examined by circular dichroism (CD) the 2°structure of AGL55 at 4°C, 25°C, and 37°C to assess whether an α‐helical structure is maintained at these temperatures. The far‐UV CD (Figure 3A) shows that, while helical structures are present at 4°C and 25°C, there is a loss of the α‐helix of AGL55 at 37°C, in agreement with other studies on a different M‐protein (Nilson et al., 1995). Upon binding to K2_hPg_, the α‐helical nature of AGL55 (Figure 3B–D) is enhanced at all three temperatures, and that the presence of K2_hPg_ does not interfere with these spectra (Figure 3E). Thus, K2_hPg_ can induce a conformational change in AGL55 that stabilizes the complex at each temperature studied.

### 3.2 Binding of K2_hPg_ to PAM_NS88.2_ results in dissociation of PAM dimers into monomers

**FIGURE 4:**
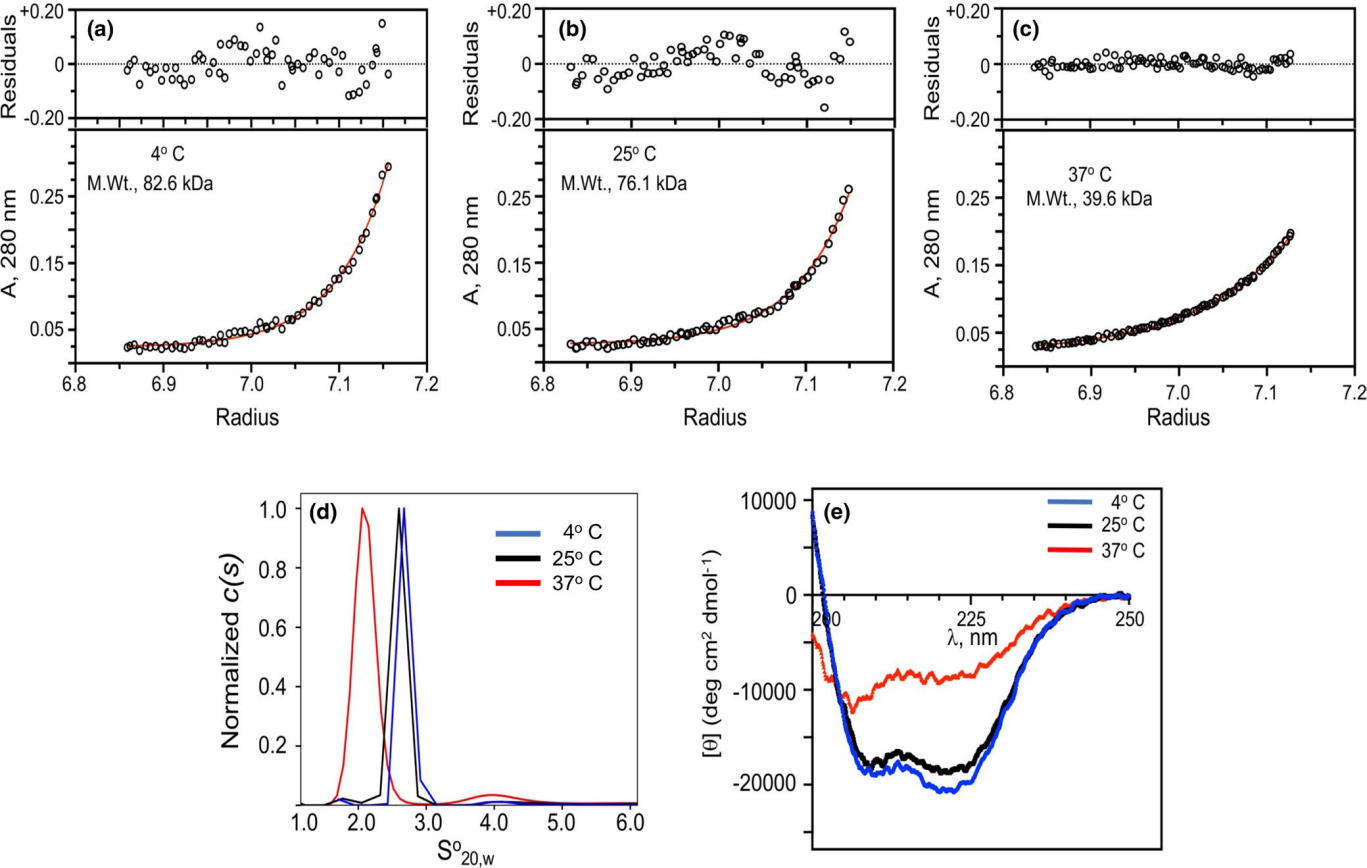
Structural comparisons of PAM_NS88.2_ at different temperatures. (A–C) *Quaternary structures*. Molecular weights of PAM_NS88.2_ at 4°C (A), 25°C (B), and 37°C (C). Equilibrium concentration gradients at the indicated temperatures were established by SE in the analytical ultracentrifuge. The initial PAM_NS88.2_ concentrations were 0.125 absorbance units at 280 nm and the rotor speed was varied at 26,100 *g*. Partial specific volumes were calculated using SEDNTERP. The values in the graph insets apply to the particular data illustrated, but the determinations were duplicated and the values fell within 5% of those shown. (D) Normalized sedimentation coefficient distributions of PAM_NS88.2_ at 4°C, 25°C, and 37°C were obtained at 6 µM PAM_NS88.2_ (based on the molecular weight of the monomer). The rotor speed was 170,600 *g* and the buffer was 50 mM Na‐phosphate/0.1 M NaCl, pH 7.4. The data are plotted as the continuous distributions, c(s), Lamm equation model against the S°_20,w_ using Sedfit. Scans were obtained every 30 s for 20 h. (E) *Secondary structures*. Far UV CD spectra were obtained at the indicated temperatures. The proportion of a‐helix is reflected by the *[θ]* values at 222 nm. In all cases, the buffer was 50 mM Na‐phosphate, pH 7.4, at the indicated temperature

We next turned our attention to the nature of the interaction of K2_hPg_ with PAM_NS88.2_. To assess the molecular species of PAM_NS288_ initially present at each of the temperatures studied, we performed AUC on PAM_NS88_._2_. For apo‐PAM_NS88.2_, at 4°C, 15°C, and 25°C (Figure 4A,B), molecular weights of 76,000–82,000 Da were obtained from data obtained at two different rotor speeds at very low concentrations (<0.2 mg/ml). This demonstrates that apo‐PAM_NS88.2_ is a dimer at 4°C–25°C. However, under similar conditions at 37°C, PAM_NS88.2_ displays a monodisperse molecular weight of ~40,000 Da (Figure 4C), which is the monomeric form of PAM_NS88.2_. These data are in general agreement with other studies demonstrating similar behavior for M1‐type M‐protein (Cedervall et al., 1995; Nilson et al., 1995) and PAM_NS88.2_ (Qiu et al., 2019) that show that a small temperature increase within a narrow range of nondenaturing temperatures destabilizes the dimeric M‐protein and a similar protein, Protein H. In concert with this, the S°_20,w_ values of dimeric PAM_NS88.2_ show single distributions of 2.6–2.8S at 4°C, 15°C, and 25°C, and was reduced to 2.1S at 37°C, also with a single S value distribution (Figure 4D). This is consistent with a molecular weight reduction to a monomer at 37°C. To assess whether the dissociation of the PAM dimers is related to the helical nature of PAM, we performed far UV CD scans of PAM at these temperatures (Figure 4e). Indeed, a large reduction of α‐helix is seen for PAM_NS88.2_ at 37°C (from originally ~65% at 4°C and 25°C to ~30% at 37°C) as reflected by a less negative mean residue ellipticity value at 222 nm, when compared to the ellipticity values at 4°C and 25°C.

**FIGURE 5:**
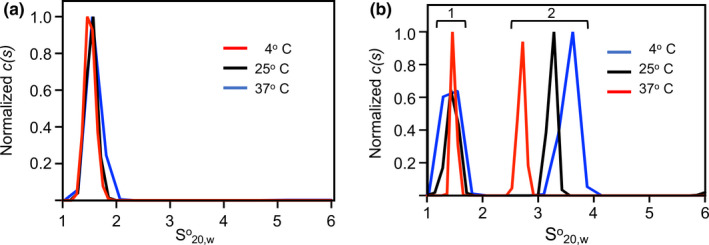
Sedimentation velocity of the K2_hPg_/PAM_NS88.2_ complex. (A) Normalized sedimentation coefficient distributions of K2_hPg_ at 4°C, 25°C, and 37°C obtained at 12 µM K2_hPg_. (B) Normalized sedimentation coefficients of the components in a mixture of 12 µM K2_hPg_/6 µM PAM_NS88.2_ (based on the molecular weight of the PAM_NS88.2_ monomer). The rotor speeds were 46,000 rpm and the buffer was 50 mM Na‐phosphate/0.1 mM NaCl, pH 7.4. The data are plotted as the continuous distribution, c(s), Lamm equation model against the S°_20,w_ using Sedfit Scans were obtained every 30 s for 20 h

Given this conclusion of a weakly associated dimer for PAM_NS88.2_, experiments were performed to determine whether K2_hPg_ binding to PAM_NS88.2_ was also capable of affecting the quaternary structure of PAM. When a mixture of 12 µM K2_hPg_/6 µM PAM_NS88.2_ (based on the monomer M. Wt. of PAM_NS88.2_) is subjected to SV experiments at each of these temperatures, two S°_20,w_ distributions are present. Figure 5A illustrates the S°_20,w_ data for the free K2_hPg_ with an S°_20,w_ of 1.6 S at 4°C, 15°C, 25°C, and 37°C. Figure 5B shows the SV data obtained for the 2/1 (m/m) mixture. Here, two S°_20,w_ clusters are observed. *Cluster 1* is the free K2_hPg_ in the mixture at all three temperatures. In *Cluster 2* the increases in S°_20,w_ values from ~2.6S (from Figure 4D) to ~3.3S at 4°C and 25°C and from ~2.1S (from Figure 4D) to ~2.7S at 37°C is due to complex formation between K2_hPg_ and PAM_NS88.2_. The data obtained at 37°C demonstrate that the monomeric form of PAM_NS88.2_, with a global reduction in α‐helix, will interact with K2_hPg_ with strong affinity. However, whether the S°_20,w_ values at 4°C and 25°C are monomers or dimers complexed with K2_hPg_ is not settled to this point. In these latter cases, the increase in S°_20,w_ values could indicate that the dimer binds two K2_hPg_ peptides or that a monomer binds a single K2_hPg_ accompanied by a less rigid coil, each of which would lead to increased S°_20,w_ values upon binding to K2_hPg_ and counteract the decrease in S°_20,w_ values resulting from dissociation of the dimeric form of PAM_NS88.2_.

**FIGURE 6:**
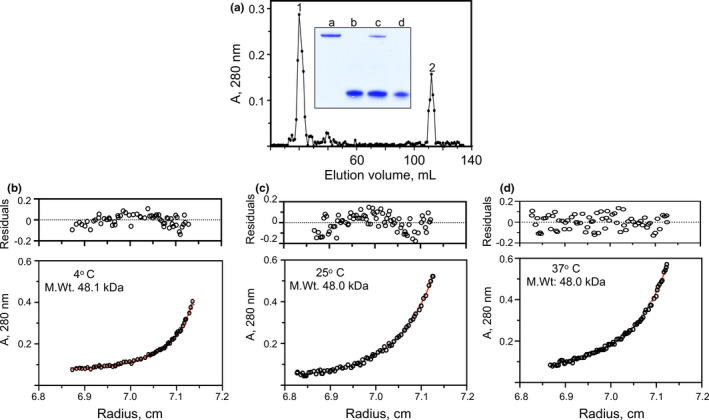
Isolation and characterization of the K2_hPg_/PAM_NS88.2_ complex. (A) Elution profile of the K2_hPg_/PAM_NS88.2_ complex on a 1 cm × 70 cm column of Sephadex G50 at 25°C. A buffer containing 50 mM phosphate/0.1 mM NaCl, pH 7.4, was used for equilibration and elution. The sample applied to the column was 27 mM K2_hPg_/18 mM PAM_NS88.2_ (based on the molecular weight of the PAM_NS88.2_ monomer). Inset. SDS‐PAGE of the pooled peaks obtained during gel filtration chromatography. *Lane a*, purified PAM_NS88.2_; *lane b*, purified K2_hPg_; *lanes c* and *d* are peaks 1 and 2, respectively, of the gel filtration chromatogram. (B–D) Equilibrium concentration gradients at the indicated temperatures were established by sedimentation equilibrium in the analytical ultracentrifuge. The temperatures were (B) 4°C, (C) 25°C, (D) 37°C. The initial PAM_NS88.2_ concentrations were 0.125 (~0.2 mg/ml) absorbance units at 280 nm and the rotor speed was 18,000 rpm. Partial specific volumes were calculated using Sednterp. The values of the graph insets apply to the particular data illustrated, but the determinations were duplicated and the values fell within 5% of those shown

Since the interaction of K2_hPg_ with PAM_NS88.2_ occurs at the low nM range, as reflected in very low k_off_ rates for hPg/PAM complexes (Qiu et al., 2018), we attempted to separate the complex of K2_hPg_ from unbound K2_hPg_ by gel filtration. A chromatogram of the elution of the 1.5:1 (m/m) K2_hPg_/PAM_NS88.2_ led to the elution of two components (Figure 6A). The 4°C, 25°C, and 37°C chromatograms were nearly identical. SDS gels (Figure 6A‐inset) show that the earliest eluting peak consists of both PAM_NS88.2_ and K2_hPg_ and the later eluting component consists of the unbound K2_hPg_. The isolated complexes were then analyzed by SE at the temperatures at which they were isolated (Figure 6B–D). The data clearly show monodisperse molecular weights of 48,000 kDa, which closely approximate the values that would be obtained by a monomeric PAM_NS88.2_ subunit in complex with a single K2_hPg_. Overall, these results demonstrate that the final complex of K2_hPg_ and PAM_NS88.2_ is composed of a monomeric PAM_NS88.2_ in a 1:1 (m:m) complex with K2_hPg_ and leads to the conclusion that dissociation of the PAM dimer to a monomer occurs when K2_hPg_ is bound to PAM_NS88.2_.

### 3.3 Thermodynamic binding of K2_hPg_ to PAM_NS88.2_

**FIGURE 7:**
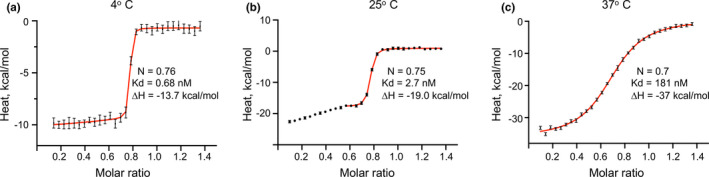
Titration of K2_hPg_ into PAM_NS88.2_ at different temperatures. Each panel shows the corresponding ITC titration curve at (A) 4°C, (B) 25°C, and (C) 37°C of K2_hPg_ into PAM_NS88.2_. The original data are shown as scattered points, and fitted curves are shown as red lines. The experimental data were best‐fit using the hetero‐association model. In each case, the fit gave the values of N, Kd, and ∆H for the K2_hPg_/PAM_NS88.2_ interaction

ITC was employed to measure the stoichiometry and dissociation constants of K2_hPg_ to PAM_NS88.2_. To more fully interpret the binding data, we also relied on biophysical data. For example, it was important to understand that the final product of the interaction was a 1:1 (m:m) complex of K2_hPg_ and PAM_NS88.2_ monomer. ITC titration data were employed at 4°C and the data are shown in Figure 7A. The molar ratio in the plot was based on the molecular weight of the PAM monomer. The curve is best‐fit in Sedphat using the simple hetero‐association model of K2_hPg_ and PAM monomer (Figure 7A). The best interpretation of the data is that K2_hPg_ first binds to the PAM dimer, which results in dissociation of the dimer and binding to the monomer. The fact that one simple titration curve is seen indicates that the binding of K2_hPg_ occurs with a similar binding constant to the dimer and monomer. The resulting Kd of 0.7 nM indicates a very tight binding of K2 to PAM. Similarly, at 37°C, PAM_NS88.2_ exists as a monomer and is also best‐fit by the simple hetero‐association model of binding (Figure 7C). Again, the titration curve is relatively simple and is deconvoluted to a Kd of 181 nM. The ΔH of −13.7 kcal/mol at 4°C is elevated to −37 kcal/mol at 37°C and this exothermic reaction is the foundation for the elevation of the Kd at this higher temperature. At 25°C, the titration curve is more complex (Figure 7b). The major final inflection of the titration curve is B to A monomer, as is evident from the biophysical data which we fit a hetero‐association model of A + B. The Kd of the single binding site increases to 2.7 nM. The initial phase of the titration (not fit) has several possible explanations, the simplest of which is that there is initial tight binding to dimer, which dissociates to a slightly weaker binding monomer (with an accompanying heat of dissociation). While this is somewhat unclear, these experiments have revealed the nature of the binding of K2_hPg_ to the PAM_NS88.2_ monomer, which is the final product of the binding. The results that we obtain for the strong hPg‐type ligand binding to PAM‐type M‐proteins at 37°C are opposite to the very weak binding of fibrinogen, IgG, and albumin binding to M1‐monomers at 37°C (Cedervall et al., 1995), thus bringing into question the biological relevance of binding of these ligands to M1 at the temperature of 37°C at which GAS infections occur.

### 3.4 Stimulation of hPg activation by SK2b is independent of PAM_NS88.2_ dimerization

From the above data, it is evident that the binding of PAM to K2_hPg_ alters the quaternary structure of PAM_NS88.2_. To examine the influence of PAM_NS88.2_ dissociation on its ability to stimulate hPg activation, especially at 37°C, a temperature at which PAM_NS88.2_ was shown to be a monomer with a significant loss in α‐helical content, hPg activation was performed in the presence of increasing PAM_NS88.2_.

**FIGURE 8:**
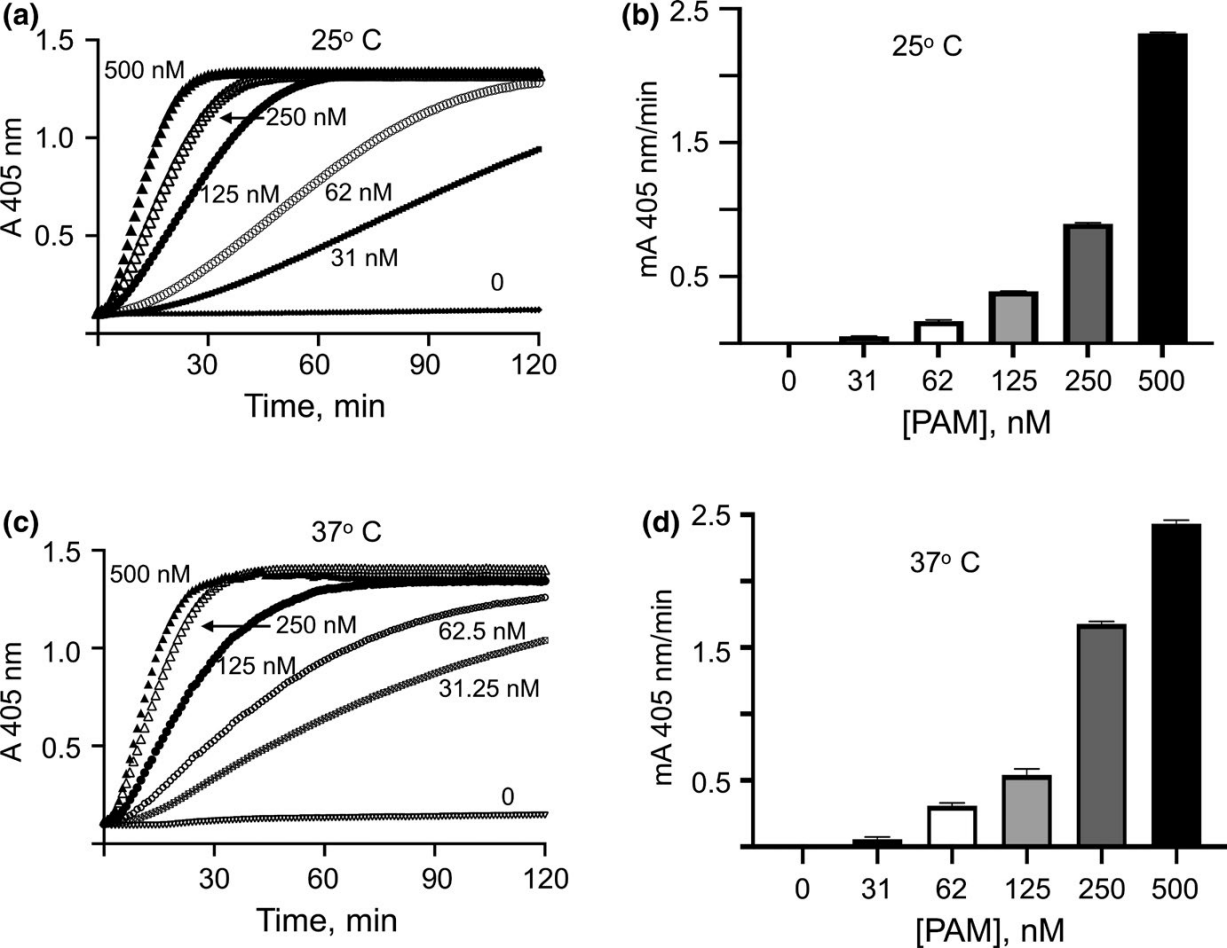
Stimulation of the SK2b‐induced activation of hPg by PAM_NS88.2_ at different temperatures. An unprocessed activation titration of hPg by SK2b at (A) 25°C and (B) 37°C. A typical assay mixture contained hPg (200 nM), various concentrations of PAM_NS88.2_ (indicated on the graphs) and S2251 (0.25 mM) in 10 mM Na‐Hepes/150 mM NaCl, pH 7.4. The reaction was accelerated by adding SK2b (5 nM). The release of p‐nitroaniline from S2251 from the continuously generated hPm was monitored at 405 nm for up to 120 min. (C) and (D) Initial velocities were calculated from plots of *A*_405nm_ vs *t* ^2^ and plotted as a function of the PAM_NS88.2_ concentrations

The data of Figure 8A–D show that SK2b which is coinherited by GAS strains (Pattern D) that express PAM (Zhang et al., 2012) is inefficient in generating plasmin (hPm) in the absence of PAM. The generation of hPm in the presence of PAM_NS88.2_ at 25°C and 37°C demonstrated that dissociation of PAM_NS88.2_ with the accompanying loss of helical content at 37°C did not affect the function of PAM_NS88.2_. Hence, dimerization is not a requirement for stimulation of hPg activation by PAM_NS88.2_.

## 4 DISCUSSION

Most molecular models for the structure of surface fibrous M‐proteins of *S*. *pyogenes* are widely accepted to be centered on a coiled‐coil α‐helical rich dimer of ~60 nm in length covalently bound at their C‐termini to the cell wall by a transpeptidation step catalyzed by sortase A. This is based on the presence of characteristic seven residue heptad repeats, a very early example being that of the coiled‐coil of α‐tropomyosin (Cohen & Parry, 1990; Parry, 1975). However, based on the data presented herein, this model requires further consideration as it applies to its biological relevance.

Secretion of M‐protein from the cytoplasm of GAS is *via* the single functional membrane microdomain, the ExPortal, which contains the SecA translocon, anionic lipids, and accessory proteins, e.g., membrane‐associated chaperones (e.g., Htra), and sortases (Rosch & Caparon, 2004). Retention of M‐protein, perhaps by the interaction of its positively charged cytoplasmic tail with anionic phospholipids in the ExPortal, allows time for unfolded M‐protein from the cytoplasm to properly mature and for enzymes such as sortase A to function to anchor M‐protein to the cell wall peptidoglycan. Thus, the ExPortal couples protein secretion with maturation. Regarding its subcellular distribution, M‐protein first appears in the septum and then becomes distributed over the entire cell surface. It thus appears that information contained in the signal sequence directs the distribution of this protein to its subcellular location (Carlsson et al., 2006).

While M‐protein secretion appears to be a highly organized series of steps, with most information required contained in its amino acid sequence, some important questions remain. Specifically, if M‐proteins exist on the surface of *S*. *pyogenes*, where does the dimerization occur? Does dimerization ever occur except *in vitro* under physiological conditions? Also, can it be assured that M‐proteins anchored to the cell wall are properly spatially organized to form dimers? Further, if monomeric M‐proteins are secreted through the ExPortal the anchoring to the cell wall should be sufficiently random that optimal dimer formation is restricted. In this case, their surface should consist of a mixture of monomers and dimers depending on their anchoring position to a large cell wall.

Some of these questions could be addressed by extrapolation of the data of this article, the most important of which is that M‐proteins do not dimerize at 37°C, the optimal temperature for these microorganisms. Thus, we suggest that monomeric M‐proteins are most likely to be present at the cell surface. With regard to Pattern D *S*. *pyogenes*, M‐protein monomers also display the optimal binding sites for hPg, a major functional protein for these strains of GAS. If dimers are present, they are dissociated by binding of hPg.

Lastly, Pattern D M‐protein monomers are functional with regard to stimulation of hPg activation by the coinherited SK2b, which does not activate hPg in solution. This step allows hPm to form and remain bound to the cell surface. The bound hPm is resistant to inactivation by its natural inhibitor, α2‐antiplasmin, and thus persists on the bacterial surface to aid in its dissemination.

In conclusion, coiled‐coil dimeric models of M‐protein have been proposed in the past, but these only appear to apply to M‐proteins in solution at temperatures up to ~25°C. While such models are highly useful for understanding the protein chemistry that allows for a‐helical coiled‐coils to form, it is unlikely that these models are appropriate for an understanding of the biology of M‐proteins on bacterial surfaces, which appear to be monomers with reduced helical content.

## 5 ETHICS STATEMENT

None required.

## CONFLICT OF INTEREST

None declared.

## AUTHOR CONTRIBUTION

**Olawole Ayinuola:** Investigation (equal); Methodology (equal). **Yetunde A. Ayinuola:** Conceptualization (equal); Data curation (equal); Formal analysis (equal); Investigation (equal); Methodology (equal). **Cunjia Qiu:** Conceptualization (equal); Data curation (equal); Formal analysis (equal); Investigation (equal); Methodology (equal). **Shaun W Lee:** Conceptualization (supporting); Funding acquisition (equal); Investigation (supporting); Methodology (supporting). **Victoria A Ploplis:** Conceptualization (supporting); Data curation (equal); Formal analysis (equal); Funding acquisition (equal); Investigation (supporting); Methodology (supporting); Project administration (supporting). **Francis J. Castellino:** Conceptualization (lead); Data curation (equal); Formal analysis (equal); Funding acquisition (lead); Investigation (lead); Methodology (equal); Project administration (lead).

## Data Availability

All data are provided in full in this article.
